# Current Synthetic Approaches to the Synthesis of Carbasugars from Non-Carbohydrate Sources

**DOI:** 10.1007/s41061-022-00370-0

**Published:** 2022-02-09

**Authors:** Alexandra Zorin, Lukas Klenk, Tonia Mack, Hans-Peter Deigner, Magnus S. Schmidt

**Affiliations:** 1 Medical and Life Sciences Faculty, Institute of Precision Medicine, Furtwangen University, Jakob-Kienzle-Str. 17, 78054 Villingen-Schwenningen, Germany; 2 EXIM Department, Fraunhofer Institute IZI Leipzig, Schillingallee 68, 18057 Rostock, Germany; 3 Faculty of Science, Associated Member of Tuebingen University, Auf der Morgenstelle 8, 72076 Tubingen, Germany

**Keywords:** Carbasugars, Carbohydrate chemistry, Synthesis, Pseudosugars

## Abstract

Carbasugars are a group of carbohydrate derivatives in which the ring oxygen is replaced by a methylene group, producing a molecule with a nearly identical structure but highly different behavior. Over time, this definition has been extended to include other unsaturated cyclohexenols and carba-, di-, and polysaccharides. Such molecules can be found in bacterial strains and the human body, acting as neurotransmitters (e.g., inositol trisphosphate). In science, there are a wide range of research areas that are affected by, and involve, carbasugars, such as studies on enzyme inhibition, lectin-binding, and even HIV and cancer treatment. In this review article, different methods for synthesizing carbasugars, their derivatives, and similar cyclohexanes presenting comparable characteristics are summarized and evaluated, utilizing diverse starting materials and synthetic procedures.

## Introduction

Carbohydrate chemistry is associated with a wide range of fields, such as organic chemistry, pharmaceuticals, medicine, and electrochemistry [1–3]. The broad range of functions of carbohydrate biomolecules, for example, the storage of energy, is made possible by the fact that carbohydrates are components of glycoproteins and glycolipids [4, 5]. As components in these types of compounds, carbohydrates are involved in an extensive range of processes, such as signalling, cell–cell communication, and molecular and cellular targeting [6]. Further biological processes, such as blood clotting and fertilization, require carbohydrates, and the biological implications of these compounds are strongly related to diseases such as cancer, diabetes, and inflammatory processes [7].

**Scheme 1:**
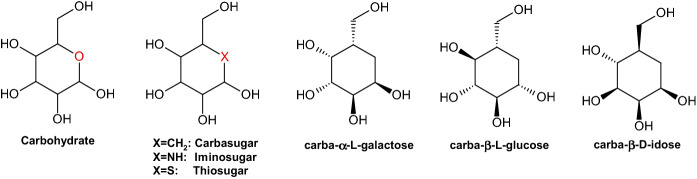
Structural similarity between carbohydrates and pseudosugars, including examples for carba-α-l-galactopyranose, carba-β-l-glucopyranose and carba-β-d-idopyranose

Based on these factors, the search for new derivatives with analogous or even improved biological properties compared with those of the natural parent structures represent a logical focus of research. The term “carbohydrate mimetic” is used frequently to refer to any carbohydrate derivative or other compound that has multiple hydroxy groups and thereby resembles a saccharide. Between 1966 and 1968, the research group of McCasland developed a series of derivatives in which the ring oxygen of a monosaccharide was replaced by a methylene group; these authors neologized the term “pseudo sugars” for this family of compounds, though such compounds are currently called “carbasugars” (Scheme 1) [8, 9]. The researchers hypothesized that the structural resemblance of carbasugars to the original sugars would enable their identification by enzymes or other biological systems in place of the related true sugars. Importantly, while guaranteeing a high similarity with true natural sugars, the slight change affords compounds with greater stability toward endogenous degradative enzymes.

McCasland et al. synthesized 5a-carba-R-dl-talopyranose (the first reported carbasugar) [8], 5a-carba-R-dl-galactopyranose [10], and 5a-carba-α-dl-glucopyranose. Notably, 7 years later, 5a-carba-R-d-galactopyranose was isolated as a truly natural product from a fermentation broth of *Streptomyces* sp. [11]. In the following four decades, the chemical, biological, and conformational aspects of carbasugars were extensively studied [12, 13], resulting in studies on enzyme inhibition, lectin-binding, and even HIV and cancer treatment [14–16].

Various strategies have been employed for the synthesis and isolation of carbasugars, mostly starting from simple monosaccharides such as glucosamine and resulting in the corresponding carbasugars [17–19]. Other approaches work from simple hexopyranoses to more complex carbafuranoses such as carbaarabinofuranosides [20, 21] or even more complex carbasugar derivatives such as fluorinated carbasugars [22]. All these methods start from simple monosaccharidic compounds and require numerous synthetic steps to produce the final product [23–29]. In past years, scientists have developed and investigated possibilities for easily accessible alternative approaches. Starting with more basic chemical structures, a wide range of different strategies have been employed to achieve the goal of synthesizing carbasugars from non-carbohydrate sources.

Natural products containing carbasugar subunits fall beyond the scope of this review and will not be dealt with here, though their application in medicinal chemistry is of course important [11]. The chemical synthesis of carbasugars and their derivatives comprises the topic of this review. There are different approaches to these compounds, which are classified broadly into two groups: synthetic methods that use non-carbohydrates as starting materials, and procedures that make use of carbohydrates as precursors [12]. In pharmaceutical production, carbasugar synthesis is not being used, as it is based most dominantly on carbohydrate sources. Due to the complexity and inefficiency of the synthesis pathways, mass production of carbasugar-based pharmaceuticals is not feasible. Thus, a new type of carbomimetic synthesis is desired in order to ensure an efficient access to carbasugar production. Therefore, in this review, we concentrate on strategies that start from non-carbohydrate sources.

## Synthesis Methods

The following sections will review six methods for synthesizing carbasugars from non-carbohydrate sources. The methods discussed are based on cylohexadiendiol, Norbornene, the Diels–Alder reaction, iodobenzene, methyl benzoate, or benzoquinone.

### Synthesis Based on Cyclohexadiendiol

**Scheme 2:**
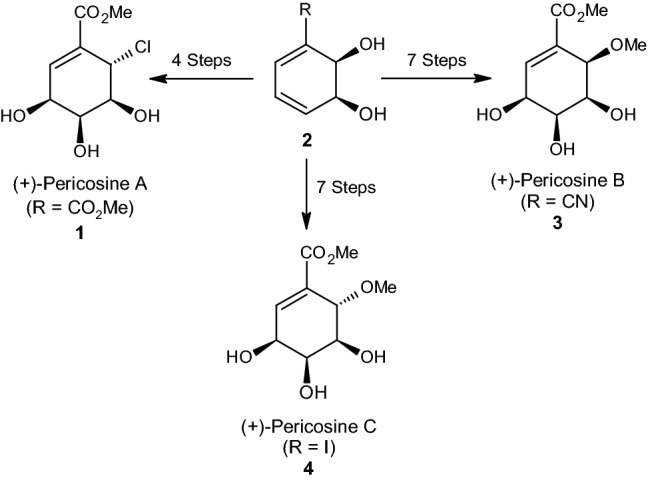
The conversion of cis-dihydrocatechols into pericosines A, B, and C

In this section, we analyze the synthesis of pseudosugars based on cyclohexadiendiol. The pericosines A (**1**), B (**3**), and C (**4**), shown in Scheme 2, occur naturally and were isolated originally from the fungus *Periconia byssoides*. This fungus can be found in the gastrointestinal tract of the sea hare *Aplysia kurodai* [30, 31]. The initial synthesis of pericosine was accomplished by Donohoe in 1998 [32]. Since then, interest in synthesizing pericosines has increased steadily.

Periscosines are known for their cytotoxicity against P388 lymphocytic leukaemia cells, antitumor activity against murine P388 cells, and selective growth inhibition against human cancer cell lines HBC-5 and SNB-75 [33]. By fulfilling these functions, pericosines may be important in treating cancer. In addition, the structural similarities between pericosines (polyhydroxylated cyclohexenes) and pseudosugars (polyhydroxylated cyclohexanes) suggest that both could be classified as carbasugars [34].

**Scheme 3:**
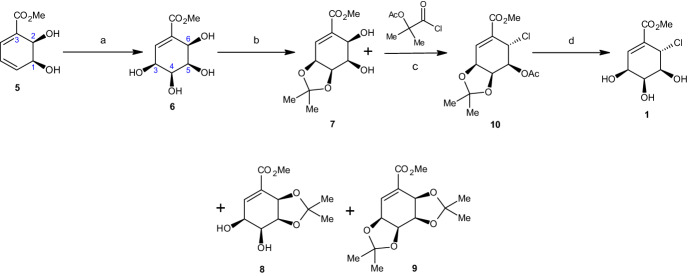
Synthesis of pericosine A. Reagents and conditions: **a** OsO_4_, Me_3_NO, CH_2_Cl_2_, rt, 12 h (70%), **b** Me_2_CO, PTSA, rt, 12 h (74%), **c** MeCN, 0 °C, 15 min, rt, 1 h (94%), **d** MeOH, cat. MeCOCl, rt, 12 h (84%)

Based on the reactions shown in Scheme 3, the dihydroxylation of cis-dihydrodiol **5**, using the Donohoe procedure [35], gave a 4:1 mixture of two cis,cis-tetraol diastereoisomers. These diastereoisomers sequentially resulted from an oxidative attack on the same face at the 5,6 and 3,4 double bonds. Following the reaction in Scheme 3, the authors were able to isolate, after chromatography, regioisomer **6** with a 70% yield [34].

Furthermore, the authors theorized that the hydroxyl group at the C6 position of compound **6** would be less reactive in the subsequent acetal reaction because the C6 hydroxyl was hydrogen-bonded to the adjacent carbomethoxy group. Following the reaction, tetraol **6** was treated at room temperature with acetone under acidic conditions, which resulted in a 7:4:9-mixture of acetonides **7**, **8**, and **9**. There was no acetal formation between the hydroxyl groups at C4 and C5. Acetonide **7** (74% overall yield) was produced by separating the mono- and bis-acetonides, repeatedly recycling the unwanted acetals **8** and **9** via hydrolysis, and reacetalizing the recovered tetraol **6** [34].

**Scheme 4:**
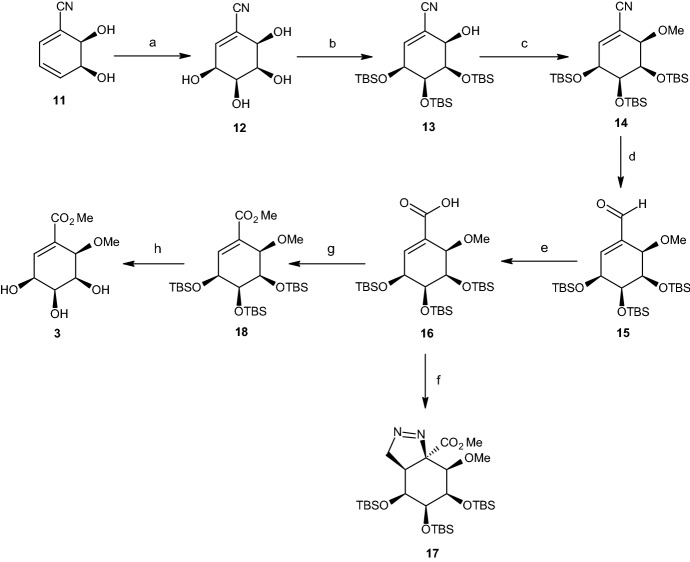
Synthesis of pericosine B. Reagents and conditions: **a** OsO_4_, Me_3_NO, CH_2_Cl_2_, rt, 48 h (55%), **b** TBSOTf, 2,6-lutidine, DMF, rt, 3 h (85%), **c** NaH, Mel, THF, rt, 40 h (62%), **d** DIBAL-H, Et_2_O, 0 °C → rt, 3 h (55%), **e** NaClO_2_, *t*-BuOH, NaH_2_PO_4_, rt, 24 h (93%), **f** CH_2_N_2_, Et_2_O, 30 min, 0 °C → rt (57%), **g** Mel, K_2_CO_3_, Me_2_CO, 35 °C, 25 min (91%), **h** TFA/H_2_O, CH_2_Cl_2_, rt, 24 h (90%)

The authors also achieved synthesis of (+)-pericosine B (**3**) after hypothesizing that this form could be synthesized from tetraol **6**. Synthesis was achieved after selective protection of the three hydroxyl groups on carbons C3, C4, and C5. To achieve the stated protection, the authors used the bulky TBS protecting group. However, the remaining hydroxyl group at C6 proved resistant to methylation under a broad range of conditions. Another way to accomplish this synthesis was to employ (1*S*,2*R*)-3-cyanocyclohex-3-ene-1,2-diol **11** bearing a CN group that was less bulky than a CO_2_Me group (Scheme 4). By following the same steps used for the reaction of cis-dihydrodiols **5** and **19**, the authors produced a 4:1 mixture of cis,cis- and cis,trans-tetraols in the dihydroxylation of diol **11**. From this, the authors isolated the major cis,cis isomer **12** with a 54% yield based on column chromatography. The reaction of tetraol **12** with tert-butyldimethylsilyl triflate produced the tri-TBS derivative **13** as the major product with 85% overall yield, together with small amounts of other unidentified inseparable isomers. The free C6 hydroxyl group of the coarse sample of silyl derivative **13** was then methylated, under mild conditions, and a purified sample of methyl ether **14** was isolated easily from the product mixture. The nitrile group in compound **14** was partially reduced to the aldehyde **15**, followed by oxidation with sodium chlorite, resulting in **16**. The authors then attempted to form the methyl ester **18** by reaction of carboxylic acid **16** with excess diazomethane. Instead of achieving the desired compound **18**, the crystalline pyrazoline cycloadduct **17** resulted from this reaction. Thus, synthesis of compound **18** is achieved by base-mediated methylation of carboxylic acid **16**, followed by acid-catalyzed removal of the TBS protecting groups, which produced (+)-pericosine B (**3**) through seven steps, with an overall yield of 12% [34].

**Scheme 5:**
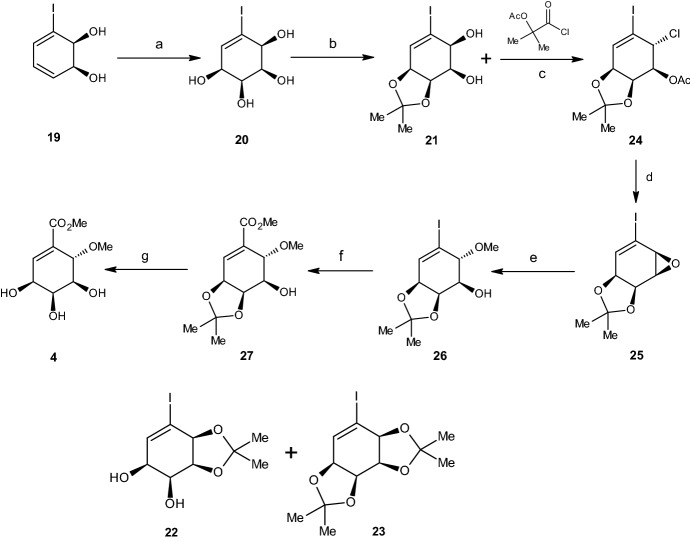
Synthesis of pericosine C. Reagents and conditions: **a** OsO_4_, Me_3_NO, CH_2_Cl_2_, rt, 3 d (71%), **b** PTSA, Me_2_CO, rt, 24 h (70%), **c** MeCN, 0 °C, 15 min, rt, 1 h (95%), **d** NaOMe, Et_2_O, 0 °C, 15 min, rt, 1 h (95%), **e** NaOMe, MeOH, rt, 12 h (68%), **f** NaOAc, MeOH, CO, Pd(OAc)_2_, rt, 12 h (89%), **g** MeOH,cat. MeCOCl, rt, 12 h (80%)

To synthesize (+)-pericosine C (**4**), as shown in Scheme 5, the authors used (1*S*,2*S*)-3-iodocyclohexa-3,5-diene-1,2-ol **19** as the starting material for the reaction. Under this alternative approach, cis-dihydrodiol **19** was dihydroxylized using the procedure in the literature (OsO4, TMNO, DCM) [34], thereby giving a 10:1 mixture of cis,cis- and cis,trans-tetraol diastereoisomers from which the required major cis,cis isomer **20** could be isolated with a 71% yield according to column chromatography. Through selective acetalization accomplished under kinetic control, along with repeated recycling of the unwanted acetals **22** and **23**, the required acetal **21** was obtained with a 70% yield. The monoacetonide **22** formed upon partial hydrolysis of bis-acetonide **23**, thereby giving access to both mono-protected forms of the tetraol (**21** and **22**). The reaction of diol **21** with 1-chlorocarbonyl-1-methylethyl acetate resulted in chloroacetate **24**. Exposing **24** to sodium methoxide and diethyl ether, provided the desired epoxide **25**. Further treatment of **25** with sodium methoxide in a methanol solution resulted in a regioselective ring opening thereby producing the methyl ether **26**. Furthermore, room temperature palladium was used to catalyze carbomethoxylation of the vinyl iodide **26** with carbon monoxide in the methanol solution, which resulted in the methyl ester **27**. Finally, the authors removed the acetonide group from ester **27** under acidic conditions in methanol, which produced (+)-pericosine C (**4**). In the authors’ opinion, this method of synthesizing (+)-pericosine C (**4**) is the most straightforward option to date, requiring only six steps from (1*S*,2*S*)-3-iodocyclohexa-3, 5-diene-1,2-ol **19**, with an overall yield of 17% [34].

In conclusion, it was demonstrated that cis-dihydrodiols derived from methyl benzoate, iodobenzene, and cyanobenzene are versatile complementary intermediates for the rapid synthesis of pericosines A, C, and B, respectively.

Additionally, the laboratories used mutant strains (e.g., UV4, 39D) of the soil bacterium *Pseudomonas putida* and *Escherichia coli*, each containing toluene dioxygenase. This factor provided access to a vast range of over 400 metabolites [34].

The strategic approach for strains to produce 2,3-trans-CHD **28** can be compared to the work performed using *Klebsiella pneumoniae* strains. Non-pathogenic *E. coli* strains are common hosts for genetic modification because of the availability of potent *E. coli* mutants and their well-established fermentation conditions. In this way, previous studies acknowledged another host for synthesizing carbasugars [34, 36].
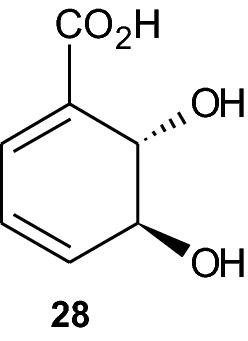


### Synthesis Based on Norbornene

**Scheme 6:**
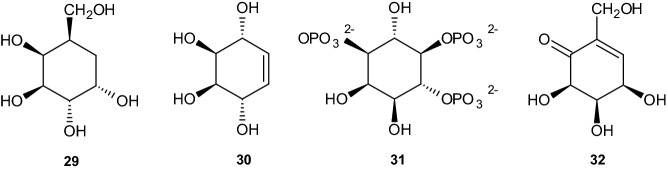
Four polyhydroxylated cylcohexenoids: pseudo-*α*-galactose (**29**), conduritol-A (**30**), myo-inositol 1,4,5-triphosphate (**31**) and gabosine-C (**32**)

In this section, we analyze carbasugar synthesis based on norbornene, hereby covering only one paper reporting this approach. The structural entities of cyclitols and the polyhydroxylated cyclohexanoids compose important segments of a wide range of natural products, like antibiotics, and they exhibit biological activity profiles extending from glycosidase inhibitors to antidiabetes and anticancer agents. Better known examples for the mentioned cyclohexitols are the carbasugars. Examples would include pseudo-alpha-galactose (**29**), conduritol-A (**30**), myo-inositol 1,4,5-triphosphate (**31**), and gabosine-C (**32**) (Scheme 6).

**Scheme 7:**
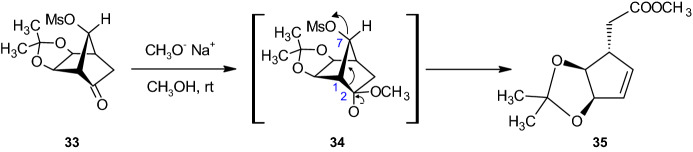
Synthesis of acetonide compound **35**

To achieve the synthesis pathway for the carbasugars, the authors first used a ‘bottom-to-top’ Grob-like fragmentation process in an easily accessible 2,7-disubstituted norbornane derivative **33** to cleave the C1–C2 bond **34**. Furthermore, the process included extraction of the five-membered ring **35** from the bridged bicyclic frame while retaining full functionalization (see Scheme 7). Taking the paper under consideration, the authors developed a beneficial method for polyhydroxylated cyclohexenoid synthesis. In the following, polyhydroxylated cyclohexenoids are obtained from the same starting materials and subsequently interchanged with functional groups, thereby showing the possibility of extracting either five- or six-membered rings from the norbornyl system [37].

**Scheme 8:**
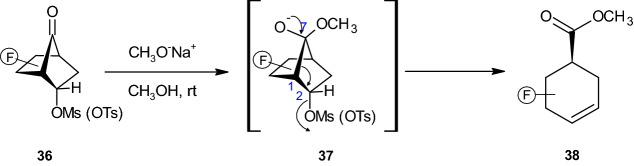
Synthesis of the embellished cyclohexanoid **38**

In the cited paper, the authors tested a new approach that involved switching the functionalities in **33** to those in **36**. By doing so, the authors arranged a “top-to-bottom” sequence involving C7–C1 bond cleavage (see **37**, Scheme 8) to deliver a functionally adorned cyclohexanoid **38** in a regio- and stereoselective manner [37].

**Scheme 9:**
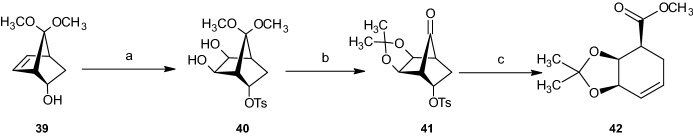
Reagents and conditions. **a** i. TsCl, Py, DMAP, DCM, rt, 90%; ii. OsO_4_, NMMO, Me_2_CO:H2O (4:1), rt, 2 d, 84%; **b** Amberlyst-15, aq. Me_2_CO, rt, 90%; **c** NaOMe, MeOH, rt, 3 h, 70%

To execute the approach shown in Scheme 8, clear access to **36** is needed. To ensure passage, the process in Scheme 9 was followed. Bicyclic alcohol **39**, available from 5,5-dimethoxy-1,2,3,4-tetrachlorocyclopentadiene and vinyl acetate, was tosylated and exposed to OsO_4_-mediated catalytic dihydroxylation to yield exo,exo-diol **40**. By designing an amberlyst mediated single-pot protection–deprotection in **40**, the authors achieved the 7-norbornanone derivative **41**. By exposing **41** to NaOMe, the authors achieved smooth top-to-bottom fragmentation, yielding the cyclohexene methyl ester as a single product. The secured stereochemistry of the six ring carbons in cyclohexenoid **42** can be further elaborated to carbasugars [37].

**Scheme 10:**
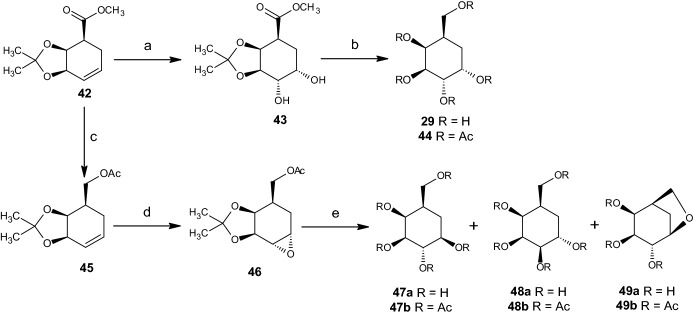
Reagents and conditions. **a** OsO_4_, NMMO, 30 h, 95%; **b** i. LAH, THF, rt, 88%; ii. Amberlyst-15, aq. MeOH, 3 h; iii. Ac_2_O, Py, 20 h, 74% (two steps); **c** i. LAH, THF, 0–5 °C, 1 h, 90%; ii. Ac_2_O, DMAP, DCM, 95%; **d** MCPBA, Na_2_CO_3_, DCM, 6 h, 65%; **e** i. cat. 70% HClO_4_, H_2_O, 30 h; ii. Ac_2_O, Py, 67% (two steps)

Dihydroxylation across the C5–C6 double bond in cyclohexenoid ester **42** occurred through addition of osmium tetroxide, producing a large yield of cis-diol **43** in a stereoselective manner (Scheme 10). Lithium aluminium hydride (LAH) reduction of the ester group of **43**, followed by acetonide deprotection provided the naturally occurring carbasugar pseudo-α-galactose (**29**). This carbasugar was characterized as penta-acetate **44**. Another method would involve the reduction of the ester group in **42** with LAH and acetylation, resulting in **45** (Scheme 10). To create **46**, the carbon–carbon double bond of **45** must be converted to an oxirane (Scheme 10). In the study, acid-catalyzed epoxide ring-opening and naturally accompanying acetonide deprotection provided a 5:2:3 mixture of pseudo-β-galactose **47a**, pseudo-α-talose **48a**, and bicyclic ether **49a**, respectively. Those components of the mixture were well separated and characterized as the corresponding acetates, **47b**, **48b**, and **49b**.

**Scheme 11:**
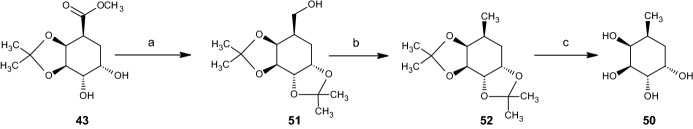
Synthesis of pseudo-*α*-fucopyranose; reagents and conditions. **a** Me_2_CO, Amberlyst-15, mol. sieves 4 Å, rt, 1 h, 85%, LAH, THF, 0 °C, 2 h, 82%, **b** TsCl, Py, DCM, rt, 94%, NaBH_4_, DMSO, 70 °C, 6 h, 72%, **c** Amberlyst-15, aq. MeOH, rt, 10 h, 75%

Finally, pseudo-α-fucopyranose (**50**), which was synthesized from ester **42**, showed a possible use as an inhibitor of fucosyltransferases. Due to this potential, the authors found another method for synthesizing **50**. Instead of using **39** as a starting point, the authors used the diol ester **43** obtained from **42**. This structure was converted into bis-acetonide and subjected to LAH reduction, yielding **51**. Next, **51** was tosylated, and reductive detosylation using sodium borohydride was carried out in DMSO. This led to installation of the *β*-methyl group and bis-acetonide **52**. Deprotection of **52** provided pseudo-α-fucopyranose (**50**), which presented spectroscopic characteristics identical to those noted in previous research [37] (Scheme 11).

### Synthesis Based on Diels–Alder Reaction

**Scheme 12:**
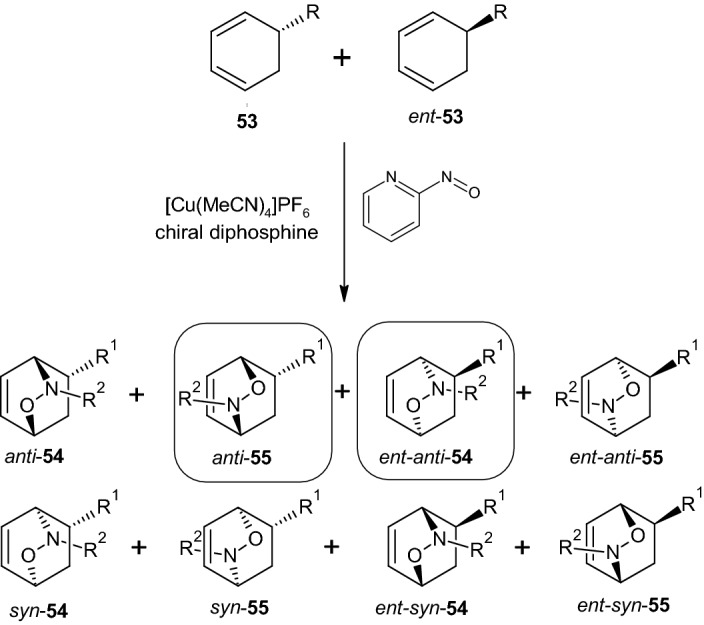
Possible isomers that can be formed through the reaction of racemic diene **53** with an aryl nitroso compound. R^1^ = phenyl, alkyl; R^2^ = 2-pyridyl

This reaction, named after its discoverers Diels and Alder from Kiel [38], involves the formation of a ring of six carbon atoms, wherein a conjugated diene and dienophile are linked. The importance of the Diels–Alder reaction is that C–C bonds can be created with high stereoselectivity. Diels–Alder reactions play an important role in the synthesis of natural substances, such as the production of steroids (e.g., the female sex hormone estradiol). In this instance, the racemic cyclohexadiene derivative **53** reacted selectively in a catalytic enantioselective Nitroso–Diels–Alder reaction to give the two main products ent-anti-**54** and anti-**55**, thereby showing the selective formation of two products from eight possible isomers (Scheme 12) [39].

The dienes used for cycloaddition were noted by the authors to be easily accessible, and the products were highlighted as valuable starting materials for the synthesis of carbasugars, such as peracetylated 2-epi-validamine (**58**, Scheme 15) [40]. It was hypothesized that divergent reactions from racemates are also possible in other Diels–Alder reactions of unsymmetrical dienophiles with racemic cyclic dienes. This approach could lead to a new concept in the field of stereoselective cycloadditions [39].

**Scheme 13:**
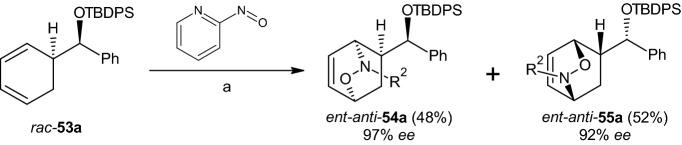
Nitroso–Diels–Alder reaction on the racemic diene **53a**. Reagents and conditions: **a** [Cu(MeCN)_4_]PF_6_/disphosphine (10 Mol-%), CH_2_Cl_2_, − 78 °C → − 20 °C

A Nitroso–Diels–Alder reaction with the highly enantiomerically enriched diene **53a** (R = (S)-CHPhOTBDPS, 98% ee) was then examined. The authors found that **53a** could be produced easily by desymmetrizing 1,4-cyclohexadiene. At the same time, the ent-compounds in Scheme 12 were not considered. The formation of adducts **54a** and **55a** occurred in CH_2_Cl_2_ in the presence of [Cu(MeCN)_4_] PF6 (10 mol%), a chiral diphosphine (10 mol%), and 2-nitrosopyridine (6 h at −78 °C and then 12 h at −20 °C). The resulting two products, ent-anti-54a and ent-anti-55a, were produced in excellent yields (column chromatography, SiO_2_) and isolated with high enantiomeric excesses (see Scheme 13) [39].

**Scheme 14:**
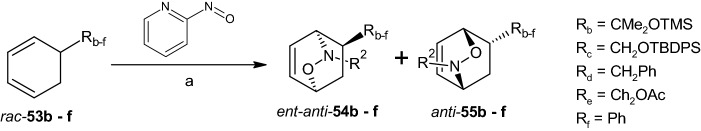
Nitroso–Diels–Alder reaction with dienes **53b–e**. Reagents and conditions: **a** [Cu(MeCN)_4_]PF_6_/disphosphine (10 mol-%), CH_2_Cl_2_, − 78°C  → − 20 °C

Next, a Nitroso–Diels–Alder reaction was carried out with the racemic dienes **53b–f** (Scheme 14). The implementation of **53b** was successful and presented excellent anti/syn-selectivity. The adducts ent-anti-54b and anti-55b were developed with high enantioselectivities. The diene **53c** showed poorer diastereoselectivity (anti/syn = 7:1) since the less sterically demanding CH_2_OTBDPS group represents a less efficient shield for the syn-side than the alpha-branched substituents. Similar results were observed for the benzyl-substituted diene **53d** and the diene **53e**, which carries an acetoxymethyl group. The best result was shown for the Ph-substituted diene **53f** (see Scheme 14) [39].

**Scheme 15:**
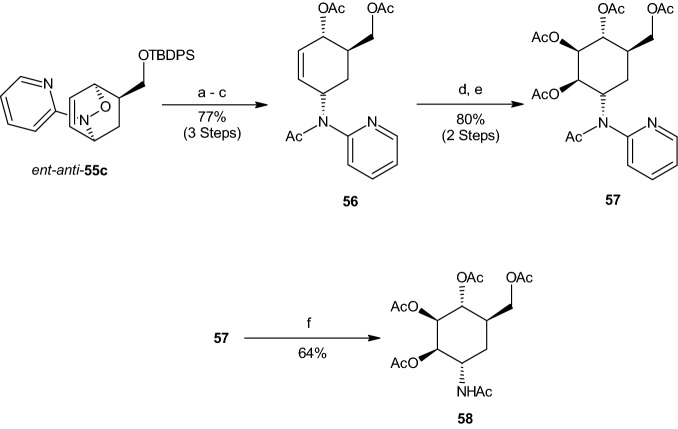
Synthesis of validamine (**58**). Reagents and conditions: **a** [Mo(CO)_6_], NaBH_4_, MeOH/H_2_O; **b** TBAF, THF; **c** 1. MeMgCl, THF, 2. AcCl; **d** K_2_OsO_2_(OH)_4_, NMO, acetone/H_2_O; **e** Ac_2_O, C_5_H_5_N. *TBAF* tetrabutylammonium fluoride, *NMO* 4-methylmorpholin-N-oxide; **f** Rh/C, H_2_ (70 bar), AcOH, 60 °C, 24 h (64%)

Finally, the method presented above was used for the synthesis of peracetylated 2-epi-validamine (**58**). Reductive N–O-bond cleavage at ent-anti-55c (89% ee), which was synthesized easily from *rac*-**53c** using an enantiomer of the Walphos ligand, with [Mo(CO)_6_] and NaBH_4_, was followed by desilylation (TBAF) and subsequent acetylation, thereby producing the cyclohexadiene **56** (Scheme 15). Diastereoselective OsO_4_-catalyzed dihydroxylation and subsequent acetylation led to the pentaacetylated carbo-sugar **57**. The N-2-pyridyl group was then cleaved using H_2_ and Rh/C, thus forming **58** [39].

**Scheme 16:**
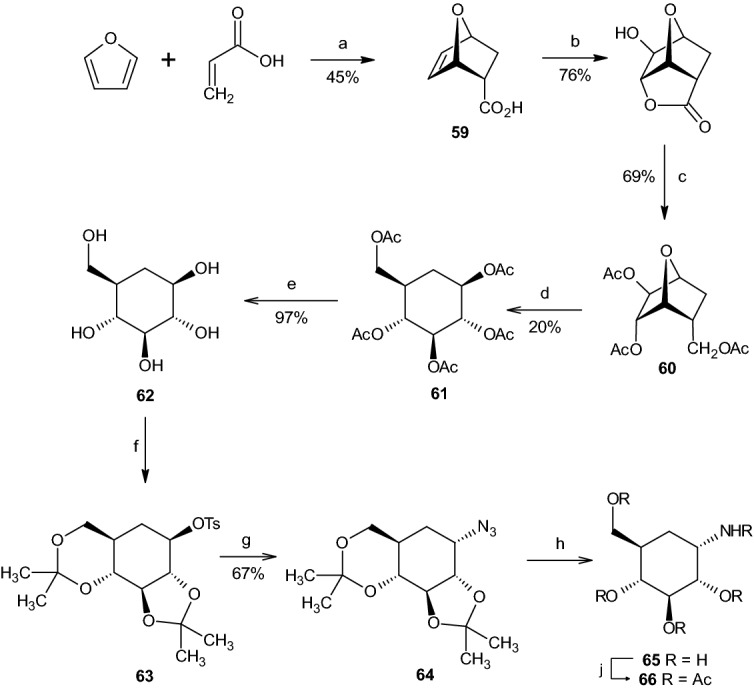
Synthesis of the racemate corresponding to validamine **65**. Reagents and conditions: **a** hydroquinone, N_2_, 105 d; **b** i) ethyl acetate, palladium black, H_2_, 30 min; ii) formic acid (95%), H_2_O_2_ (30%), 45 °C, 5 min; **c** i) LiAlH_4_, THF, H_2_O, 5 °C, 15 min; ii) Ac_2_O, C_5_H_5_N, Al_2_O_3_, CHCl_3_; **d** AcOH, H_2_SO_4_, 22 h, Ac_2_O; **e** MeONa, 70 °C, 1 h, Amberlite IR-120(H^+^); **f** i) 2,2-DMP, DMF, *p*-TSA, 60 °C, 3 h; ii) sodium hydrogencarbonate, *p*-TsCl, 2 d; **g** NaN_3_, DMF, 2 h; **h** i) EtOH, Raney Ni, H_2_, 18 h; ii) HCl, 80 °C, 1 h, IRA-400(OH^−^); **j** Ac_2_O, C_5_H_5_N, 2 d

Another approach for the synthesis of a racemate corresponding to validamine **65** was developed by Suami et al. [41, 42], whose approach was based on the Diels–Alder addition of furan to acrylic acid, resulting in oxanorbornene **59** (Scheme 16). Through hydrogenation in ethyl acetate and oxidation utilizing H_2_O_2_, an intermediate compound was achieved in 76% yield. Reduction and acetylation gave 69% of oxanorbornane **60**, followed by acetolysis with AcOH and H_2_SO_4_, giving protected 5a-carba-β-dl-glucopyranose **61** (20% yield). Deprotection of **61** resulted in 5a-carba-β-dl-glucopyranose (**62**). Reaction of compound **62** with DMP in dimethylformamide (DMF) in the presence of *p*-TSA and subsequent crystallization resulted in an inseparable 2:3 crystalline sulfonate mixture of compound **63** and 3-*p*-toluenesulfonate. Sodium azide in DMF caused creation of a new hexane compound from **63**, while preserving the 3-*p*-toluenesulfonate (11%). Evaporation gave 67% of the new azide compound **64**. Reduction with H_2_ in the presence of *Raney* nickel in ethanol, followed by acid hydrolysis of the protective groups resulted in the racemate corresponding to validamine **65**. The racemate was isolated as penta-N,O-acetyl-dl-validamine (28%, Scheme 16) [41, 42].

### Synthesis Based on Iodobenzene

The next major sources of non-carbohydrate sources for carbasugar synthesis are monosubstituted benzenes. These include regular benzene, methyl benzoate, toluene, and other derivatives of the benzene molecule [43–45]. Here, we outline the various synthetic pathways based on iodobenzene, while reactions based on methyl benzoate are described in the next section.

**Scheme 17:**
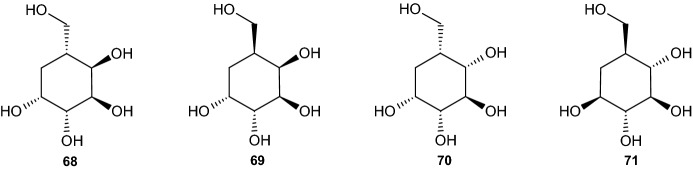
Four carbasugar isomers

The research of Boyd et al. [43] precisely describes the synthesis of four 5-(hydroxymethyl)cyclohexane-1,2,3,4-tetraol stereoisomers and their penta-acetylated analogues based on iodobenzene (1*S*,2*S*)-*cis-*dihydrodiol **67**. By using the (1*R*,2*R*)-*cis-*dihydrodiol metabolite of iodobenzene, the same reaction processes yielded four additional stereoisomeric forms [43]. In the following section, we explain the different reactions used to obtain the four carbasugars presented in Scheme 17, carba-β-d-altropyranose (**68**), carba-α-l-galactopyranose (**69**), carba-β-d-idopyranose (**70**), and carba-β-l-glucopyranose (**71**), including their penta-acetate derivatives (not shown) [43].

#### Synthesis of Carba-β-d-Altropyranose and Carba-α-l-Galactopyranose

**Scheme 18:**
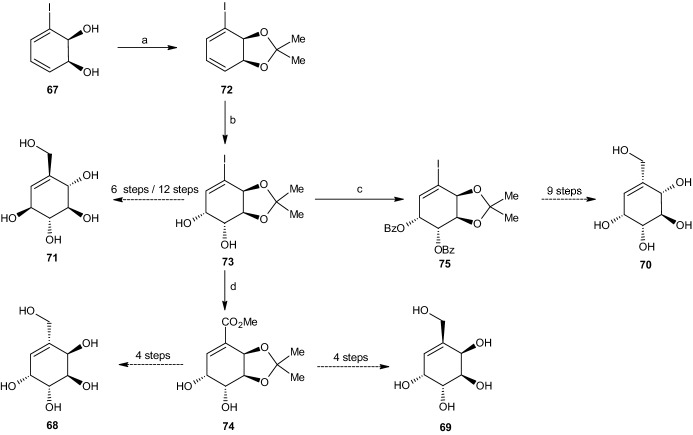
Synthesis pathways summarized. Reagents and conditions: **a** 2,2-DMP, 90% yield, **b** OsO_4_, Me_2_CO, H_2_O, 87% yield, **c** BzCl, C_5_H_5_N, 95% yield, **d** Pd(OAc)_2_, CO, NaOAc, MeOH, 81% yield

The reaction processes for all four carbasugars are based on iodobenzene cis-dihydrodiol **67** (Scheme 18). The synthesis of iodobenzene cis-dihydrodiol from iodobenzene through the use of *Pseudomonas putida* was clearly reported by Derek et al. [46] and will not be elaborated. The first reaction step involved the addition of a protecting group at the two hydroxyl substituents. This was performed via the addition of 2,2-dimethoxypropane, thereby forming an acetonide protecting group and resulting in a 98% yield of the (3a*S*,7a*S*)-acetonide derivative **72** (Scheme 18). This step was followed by dihydroxylation through the use of catalytic amounts of osmium tetroxide in acetone and water in the presence of *N*-methylmorpholine *N*-oxide. This process resulted in the (3a*S*,4*R*,5*R*,7a*S*)-diol acetonide isomer **73** with a yield of 87%. Under a carbon monoxide atmosphere with sodium acetate and methanol, the (3a*R*,6*R*,7*R*,7a*S*)-*α*,*β*-unsaturated ester **74** was created with a yield of 81% after being catalyzed through palladium(II) acetate [43].

**Scheme 19:**
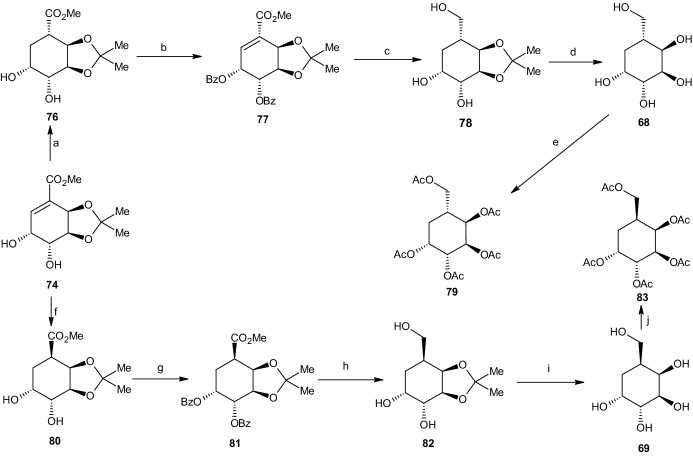
Synthesis of carba-β-d-altropyranose (**68**) and carba-α-l-galactopyranose (**69**). Reagents and conditions: **a** H_2_, Rh/Al_2_O_3_, 35% yield, **b** BzCl, C_5_H_5_N, 80%/28% yield, **c** LiAlH_4_, 74% yield, **d** TFA, 90% yield, **e** Ac_2_O, C_5_H_5_N, 78% yield, **f** Rh/AL_2_O_3_, H_2_, 65% yield, **g** BzCl, C_5_H_5_N, 86%/56% yield, **h** LiAlH_4_, 76% yield, **i** TFA, 81% yield, **j** Ac_2_O, C_5_H_5_N, 84% yield

Under a pressure of 55 psi, catalytic hydrogenation occurred, resulting in an inseparable mixture consisting of the two epimers (3a*R*,4*S*,6*R*,7*R*,7a*S*) **76** and (3a*R*,4*R*,6*R*,7*R*,7a*S*) **80** (Scheme 19), with yields of 35% and 65%, respectively. The next step involved converting both isomers into their dibenzoate derivatives to enable chromatographic separation by preparative thin layer chromatography (PTLC), yielding about 80% and 86%, respectively (or 28% and 56% relative to the catalytic hydrogenation step). After separation of the mixture, the three ester groups of the (3a*R*,4*S*,6*R*,7*S*,7a*R*)-dibenzoate compound **77** were reduced using LAH, yielding (3a*S*,4*R*,5*R*,7*R*,7a*R*)-acetonide triol **78** (74%). Using acid-catalyzed deprotection with trifluoroacetic acid, the reactions yielded the desired carba-β-d-altropyranose (**68**, 90%), which was then purified via charcoal/celite chromatography. The carbasugar was further transformed into its penta-acetate derivative **79** through the use of acetic anhydride and pyridine. The reaction process for carba-α-l-galactopyranose (**69**) occurred under the same conditions.

After separation through PTLC, the other compound (3a*R*,4*R*,6*R*,7*S*,7a*R*)-dibenzoate **81** was treated with LAH, forming (3a*S*,4*R*,5*R*,7*S*,7a*R*)-acetonide triol **82** in 76% yield (Scheme 19). Removal of the protecting group, purification, and conversion to the penta-acetate followed the same reaction pathway as that for carba-β-d-altropyranose, yielding 81% of carba-l-galactopyranose (**69**) and 84% of its penta-acetate derivative **83** [43].

#### Synthesis of Carba-β-d-Idopyranose

**Scheme 20:**
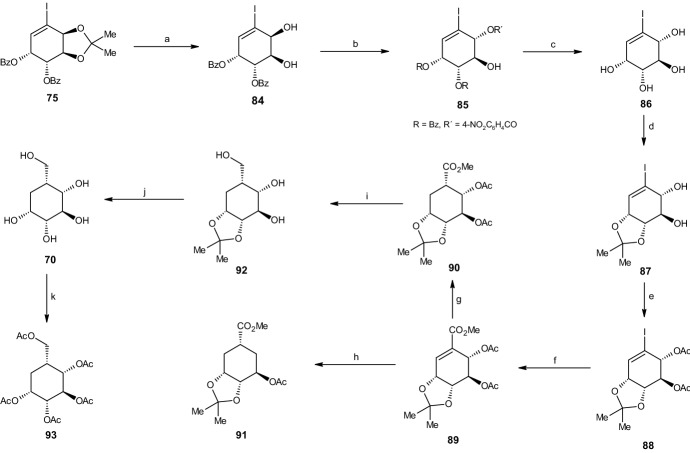
Synthesis of carba-β-d-idopyranose. Reagents and conditions: **a** HCl, MeOH, 90% yield, **b** PPh_3_, DEAD, 4-NO_2_C_6_H_4_CO_2_H, 70% yield, **c** NaOH, MeOH, 87% yield, **d** 2,2-DMP, p-TSA, 89% yield, **e** Ac_2_O, C_5_H_5_N, 95% yield, **f** CO, Pd(OAc)_2_, NaOAc, MeOH, 87% yield, **g** Rh/Al_2_O_3_, H_2_, EtOH, 40% yield, **h** Rh/Al_2_O_3_, H_2_, EtOH, 60% yield, **i** LiAlH_4_, THF, 81% yield, **j** HCl, MeOH, 79% yield, **k** Ac_2_O_3_, C_5_H_5_N, 97% yield

The synthesis of carba-β-d-idopyranose (**70**) was more challenging. With the synthesis of carba-β-d-altropyranose (**68**) and carba-α-l-galactopyranose (**69**), the relative configuration of C2 and C3 was identical to those at the iodobenzene dihydrodiol **67**. However, with carba-β-d-idopyranose **70**, the relative configuration at C2 and C3 was trans, thus requiring inversion at C2. The reaction began by treating the previously synthesized (3a*S*,4*R*,5*R*,7a*S*)-diol acetonide isomer **73** with benzoyl chloride and pyridine, giving a 95% yield of (3a*R*,4*S*,5*R*,7a*S*)-dibenzoate **75** (Scheme 20). This compound was deprotected by hydrochloric acid in methanol, resulting in a 90% yield of dibenzoate **84**. Using triphenylphosphine, diethyl azodicarboxylate (DEAD), and 4-nitrobenzoic acid, with Mitsunobu inversion occurring at the targeted allylic position, the dibenzoate resulted in (1*R*,4*R*,5*S*,6*S*)-*p*-nitrobenzoate **85** (70% yield). By utilization of sodium hydroxide in methanol, esters R and R′ (see Scheme 20) were hydrolyzed, resulting in (1*R*,2*R*,3*S*,4*R*)-tetraol **86** with a yield of 87%. Subsequently, a protective acetonide group was added through the addition of 2,2-DMP and *p*-toluenesulfonic acid (*p*-TSA), yielding 89% of (3a*S*,4*R*,5*R*,7a*R*)-acetonide **87**, which was then diacetylated at the two trans hydroxyl groups, forming (3a*R*,4*S*,5*R*,7a*R*)-diacetate **88** (95% yield) with Ac_2_O and pyridine. Carbonylation with CO, catalyzed by palladium(II) acetate and sodium acetate in methanol, was used to replace the iodine with a carbomethoxy group, thereby forming the (3a*R*,6*S*,7*S*,7a*R*)-triester **89**. Removing the alkene while also ensuring the desired relative configuration via catalytic hydrogenation (similar to Scheme 19) through Rh/Al_2_O_3_ and H_2_ in ethanol yielded 40% of (3a*R*,5*S*,6*S*,7*S*,7a*R*)-triester **90**. However, due to the competing hydrogenolysis, there was also a 60% yield of an undesired (3a*R*,5*S*,7*R*,7a*R*)-diester **91** (Scheme 20), which was separated easily by chromatography. The reaction continued with triester **90**, which was deprotected via the removal of the three ester groups produced by LAH and tetrahydrofuran (THF), giving (3a*R*,4*R*,5*S*,6*R*,7a*R*)-triol **92** (81% yield), followed by the removal of the acetonide group through HCl in methanol, yielding 79% of carba-β-d-idopyranose (**70**). Similar to both preceding reactions, the carbasugar was transformed into its penta-acetate derivative **93**, again through the use of Ac_2_O and pyridine, with a yield of 97% [43].

#### Synthesis of Carba-α-l-Glucopyranose

**Scheme 21:**
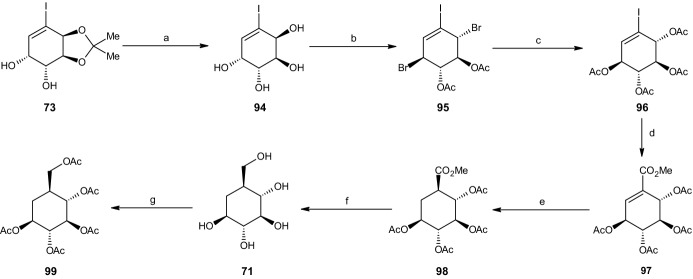
Synthesis of carba-β-l-glucopyranose. Reagents and conditions: **a** HCl/MeOH, 85% yield, **b** AcOCMe_2_COBr, 87% yield, **c** AgOAc/AcOH/Ac2O, 77% yield, **d** Pd(OAc)_2_, CO, NaOAc, THF, H_2_O, 73% yield, **e** Rh/Al_2_O_3_, H2, 80% yield, **f** LiAlH_4_, 12% yield, **g** Ac_2_O, 95% yield

The reaction pathway for the final carbasugar carba-α-l-glucopyranose (**71**) started with (3a*S*,4*R*,5*R*,7a*S*)-diol acetonide isomer **73**, synthesized under the carba-b-d-altropyranose pathway (Scheme 18). Again, the trans-configuration at C2 and C3 differed from that of iodobenzene cis-dihydrodiol **67**, as well as that at C4 and C5, requiring inversion at C2 and C5. The reaction started by deprotecting compound **73** using HCl in methanol (Scheme 21), yielding 85% of (1*R*,2*R*,3*S*,4*S*)-anti-tetraol **94**, followed by treatment with 1-bromocarbonyl-1-methylethyl acetate, which produced the desired inversion at C2 and C5 and yielded 87% of (1S,2R,5S,6S)-dibromo diacetoxy derivative **95**. Utilizing Woodward–Winstein conditions [47], silver acetate, acetic acid, and Ac_2_O yielded 77% (1*R*,2*S*,5*R*,6*S*)-tetra-acetate **96**, preserving all four desired chiral configurations. The addition of palladium(II) acetate, NaOAc, THF, and water under a carbon monoxide atmosphere was performed to replace the iodine with a carbomethoxy group, yielding 73% of unsaturated (3*S*,4*R*,5*R*,6*S*)-tetra-acetate **97**, followed by catalytic hydrogenation, which resulted in saturated (1*R*,2*S*,3*R*,4*R*,5*S*)-tetra-acetate **98** with a yield of 80% under similar conditions to those employed in earlier synthesis pathways, using Rh/Al_2_O_3_ and H_2_. Reducing the tetra-acetate with LAH yielded 12% of carba-α-l-glucopyranose (**71**). Due to this surprisingly low yield, another synthesis pathway using a higher step count was developed. Similar to all three other carbasugars, (1*S*,2*S*,3*R*,4*R*,5*S*)-penta-acetate **99** was formed under the same conditions, yielding 95% (Scheme 21) [43].

#### Alternate Synthesis of Carba-β-l-Glucopyranose

As noted earlier, the previously presented synthesis pathway for carba-β-l-glucopyranose did not yield a suitable amount of the desired carbasugar. Thus, a longer yet more efficient synthesis pathway was developed.

**Scheme 22:**
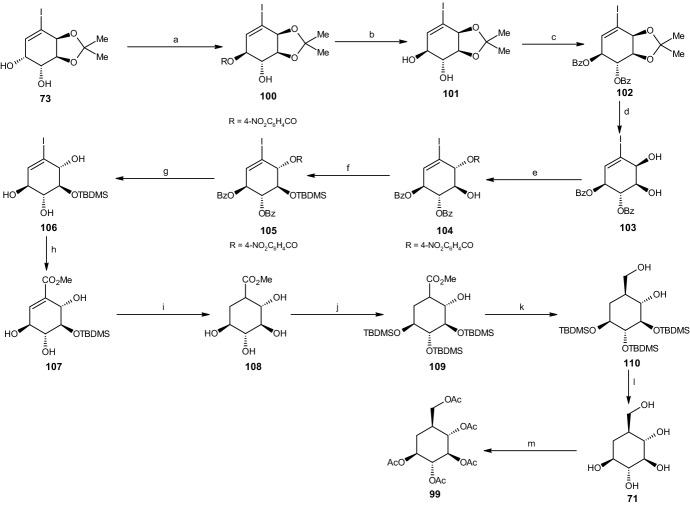
Alternate synthesis of carba-β-l-glucopyranose. Reagents and conditions: **a** PPh_3_, DEAD, 4-NO_2_C_6_H_4_CO_2_H, 80% yield, **b** K_2_CO_3_, MeOH, 82% yield, **c** BzCl, C_5_H_5_N, 93% yield, **d** HCl, MeOH, 86% yield, **e** PPh_3_, DEAD, 4-NO_2_C_6_H_4_CO_2_H, 80% yield, **f** TBDMSOTf, 93% yield, **g** NaOH, MeOH, 82% yield, **h** CO, Pd(OAc)_2_, NaOAc, THF, H_2_O, 69% yield, **i** Rh/Al_2_O_3_, H_2_, 80% yield, **j** TBDMSOTf, 95% yield, **k** LiAlH_4_, 82% yield, **l** TBAF, THF, 78% yield, **m** Ac_2_O, C_5_H_5_N, 97% yield

The reaction started with the same reactant used in 2.4.3: (3a*S*,4*R*,5*R*,7a*S*)-cis-diol acetonide **73**. Utilizing Mitsunobu inversion conditions similar to those presented in Scheme 20, inversion occurred at C5, yielding 80% (3a*S*,4*S*,5*S*,7a*S*) 4-nitrobenzoate **100** (Scheme 22). Through the use of potassium carbonate in methanol, the nitro benzoate compound **100** was transformed into (3a*S*,4*R*,5*S*,7a*S*)-trans-diol acetonide **101** (82% yield), in which the two trans hydroxyl groups were protected by the benzoyl groups, yielding 93% (3a*S*,4*S*,5*R*,7a*S*)-trans-dibenzoate acetonide **102**. An acid-catalyzed reaction led to removal of the acetonide group from compound **102**, forming (1*S*,4*S*,5*R*,6*S*)-cis-diol dibenzoate **103** with a yield of 86%. A second Mitsunobu reaction was then required to achieve the final relative configuration of all five carbon atoms. This occurred under the same conditions reported previously, resulting in inversion at C2 and yielding 80% of the (1*R*,4*S*,5*S*,6*S*)-triester compound **104**. The hydroxyl group at C3 was protected by the addition of trifluoromethanesulfonic acid tert-butyldimethylsilyl ester (TBDMSOTf), giving a 93% yield of (1*R*,4*S*,5*R*,6*S*)-TBDMS ether **105**, followed by deprotection of the three remaining hydroxyl groups using NaOH in methanol, thereby yielding 86% of (1*S*,2*R*,3*S*,4*R*)-triol **106** (Scheme 22). The next step involved substitution of the iodine with a carbomethoxy group using similar conditions to those employed in previous schemes, thus forming an unsaturated (3*S*,4*R*,5*R*,6*S*)-ester **107** with a yield of 69%. This ester compound was hydrogenated into the saturated (1*R*,2*S*,3*R*,4*R*,5*S*)-ester **108** with a yield of 80% and trans configuration between the carbomethoxy and adjacent hydroxyl group. Through the addition of TBDMSOTf, (1*S*,2*S*,3*R*,4*R*,5*S*)-tri-TBDMS ester **109** was achieved. Similar to previously described schemes, the addition of LAH resulted in reduction of the carbomethoxy group to a hydroxyl methyl group, yielding compound (1*S*,2*R*,3*R*,4*S*,5*S*)-tri-TBDMS ether **110**.

Deprotection in HCl and methanol using tetra-*n*-butylammonium fluoride (TBAF) and THF resulted in a 78% yield of the desired carba-*β*-l-glucopyranose (**71**). As with all previous reactions, the carbasugar was purified using charcoal/Celite and further characterized as its penta-acetate derivative **99** (Scheme 22) [43].

#### Synthesis of (−)-Gabosine A

**Scheme 23:**
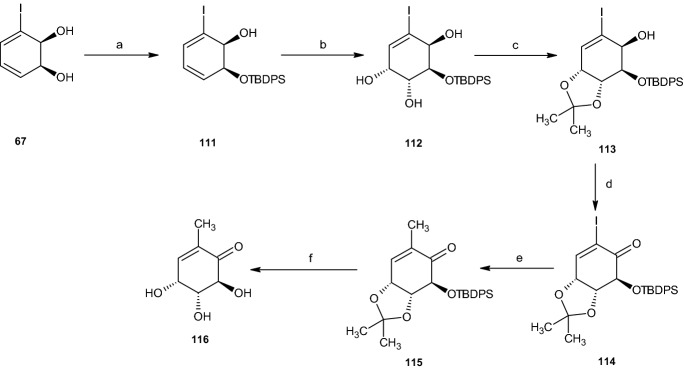
Synthesis of (−)-gabosine A (**116**). Reagents and conditions: **a** TBDPSCl, imidazole, CH_2_Cl_2_, **b** OsO_4_, NMO, acetone-H_2_O, **c** 2,2-DMP, *p*-TSA, Et_3_N, **d** COCl_2_, DMSO, Et_3_N, **e** MeMgCl, FeCl_3_, NMP, THF, **f** HCl, MeOH, (Me_2_N)_3_S^+^F_2_SiMe_3_^−^, THF

Based on iodobenzene (1*S*,2*S*)-cis-dihydrodiol **67**, Banwell et al. [48] presented a pathway that provides (−)-gabosine A (**116**) over six steps (Scheme 23). The reaction started by protecting compound **67** at the less sterically hindered hydroxyl group at C1 under a nitrogen atmosphere, thus forming TBDPS-ether **111**. Utilizing UpJohn dihydroxylation conditions [49], two hydroxyl groups were formed at the non-halogenated carbon–carbon double bond, yielding triol **112**. The addition of an acetonide occurred under known conditions in the presence of triethylamine, thereby forming acetonide compound **113**. This compound was oxidized under Swern conditions, yielding the ketone **114**, followed by replacement of the iodine with a methyl group, utilizing iron-catalyzed reactions developed by Cahiez and Avedissian [50].

Deprotection under acidic conditions and THF gave a 1:2 mixture of (−)-gabosine A (**116**) and its 6-TBDPS ether derivative, which was then treated with THF and tris(dimethylamino)sulfonium difluorotrimethylsilicate (TASF), to remove the silyl ether group and form the desired (−)-gabosine A (**116**, Scheme 23) [48].

### Synthesis Based on Methyl Benzoate

As stated in the previous section, various different types of benzene derivatives are used for carbasugar synthesis. This section explores methyl benzoate as a reactant. Similarly, methyl benzoate was converted into its cis-dihydrodiol metabolite, thereby ensuring the same skeletal structure as mono carbasugars along with the desired relative configuration at C2 and C3 to synthesize three more sugar analogues.

**Scheme 24:**
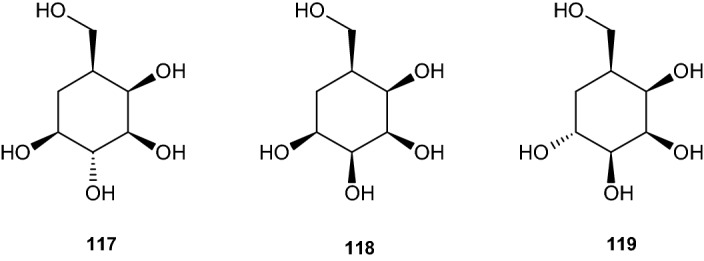
Three carbasugar isomers

The work of Boyd et al. explored the synthesis of three more carbasugars, carba-β-l-galactopyranose (**117**), carba-β-l-talopyranose (**118**), and carba-α-l-talopyranose (**119**, Scheme 24), based on the same starting material presented in the previous paragraph, iodobenzene cis-dihydrodiol **67**, which was carbonylated using palladium(II) acetate and NaOAc·3H_2_O in methanol under a carbon monoxide atmosphere to yield the (1*S*,2*R*)-cis-dihydrodiol derivative **120** of methyl benzoate [51]. This cis-dihydrodiol was the starting point for all three synthesis pathways.

#### Synthesis of Carba-β-l-Galactopyranose

**Scheme 25:**
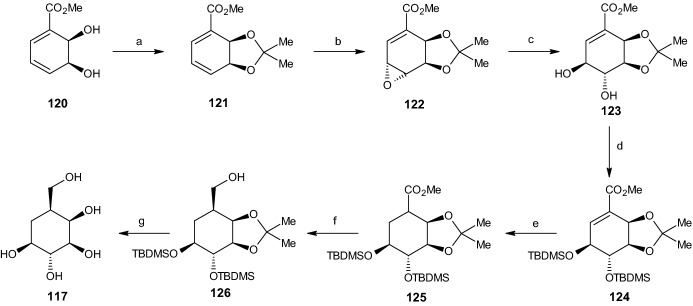
Synthesis of carba-β-l-galactopyranose. Reagents and conditions: **a** 2,2-DMP, *p*-TSA, 93% yield, **b** MCPBA, CH_2_Cl_2_, 77% yield, **c** ^*t*^BuOH, H_2_O, pH 8 buffer, 70% yield, **d** TBDMSOTf, Et_3_N, CH_2_Cl_2_, 82% yield, **e** Rh/Al_2_O_3_, H_2_, EtOH, 62% yield, **f** LiAlH_4_, Et_2_O, 84% yield, **g** MeOH, HCl, 84% yield

The synthesis started by protecting the previously created cis-dihydrodiol derivative **120** with DMP and p-TSA, yielding 93% of the acetonide compound **121** (see Scheme 25). Epoxidation occured at C5 and C6 via epoxidation using meta-chloroperoxybenzoic acid (MCPBA) in dichloromethane to yield epoxide **122** (77% yield). Using tert-butanol in water with a pH 8 buffer led to ring opening at the epoxide, yielding 70% of trans-dihydrodiol **123**. This compound was protected by tert-butyldimethylsilyl trifluoromethanesulfonate (TBDMSOTf) at C4 and C5 to give the di-TBDMS derivative **124** and hydrogenation similar to the previous reactions catalyzed by Rh/Al_2_O_3_ yielded 82% of the saturated compound **125**. This was followed by treatment with LAH in Et_2_O, resulting in 84% of the protected carba-β-l-galactopyranose **126**. The compound was deprotected under acidic conditions, yielding 84% of carba-β-l-galactopyranose (**117**) [51].

#### Synthesis of Carba-β-l-Talopyranose

**Scheme 26:**
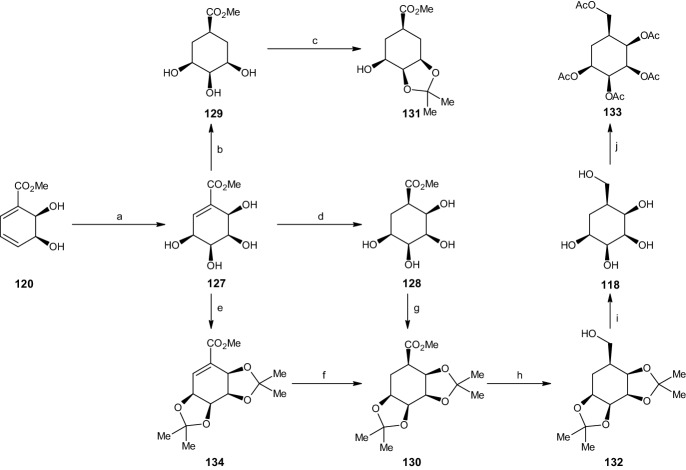
Synthesis of carba-β-l-talopyranose. Reagents and conditions: **a** OsO_4_, TMANO, CH_2_Cl_2_, 70% yield, **b** Rh/Al_2_O_3_, H_2_, EtOH, 30% yield, **c** 2,2-DMP, *p*-TSA, 25% yield, **d** Rh/Al_2_O_3_, H_2_, EtOH, 70% yield, **e** 2,2-DMP, *p*-TSA, 80% yield, **f** Rh/Al_2_O_3_, H_2_, EtOH, 91% yield, **g** 2,2-DMP, *p*-TSA, 59% yield, **h** LiAlH_4_, Et_2_O, 76% yield, **i** TFA, THF, H_2_O, 86% yield, **j** Ac_2_O, C_5_H_5_N, 85% yield

The next reactions started with the previously synthesized cis-dihydrodiol methyl benzoate derivative **120** (Scheme 26). The addition of osmium tetroxide with trimethylamine-*N*-oxide in a dichloromethane solution resulted in a 70% yield of the tetraol compound **127**. Catalytic hydrogenation resulted primarily in the saturated tetraol compound **128**, while also yielding 30% of the achiral meso-triol **129**. This mixture was inseparable under charcoal/Celite chromatography and was treated with 2,2-DMP and p-TSA, resulting in a mono acetonide **131**/bis-acetonide **130** mixture, which was then separated using flash-column chromatography to yield 59% of the bis-acetonide compound **130**.

An alternative synthesis route was also explored. This route began with compound **127** and results in the same saturated bis-acetonide compound **130**. This synthesis was achieved by changing the order of the two reactions (hydrogenation and protecting). This process started by protecting all four hydroxyl groups with an acetonide using 2,2-DMP and p-TSA to yield 80% of another bis-acetonide compound **134** and was followed by catalytic hydrogenation utilizing the previously presented conditions. This process afforded 91% of the desired saturated bis-acetonide while completely suppressing the competing hydrogenation reaction (i.e., **127** → **128**/**129**). Both remaining steps were similar to all previous reactions, starting with reduction of the carbomethoxy group function to a hydroxymethyl group (Scheme 26) using LAH in Et_2_O to give alcohol compound **132** (76% yield) and deprotection using TFA in aqueous THF to yield 86% of carba-*β*-l-talopyranose (**118**). The carbasugar was then converted to its penta-acetate derivative **133** using A_c2_O and pyridine (85% yield) for characterization [51].

#### Synthesis of Carba-α-l-Talopyranose

**Scheme 27:**
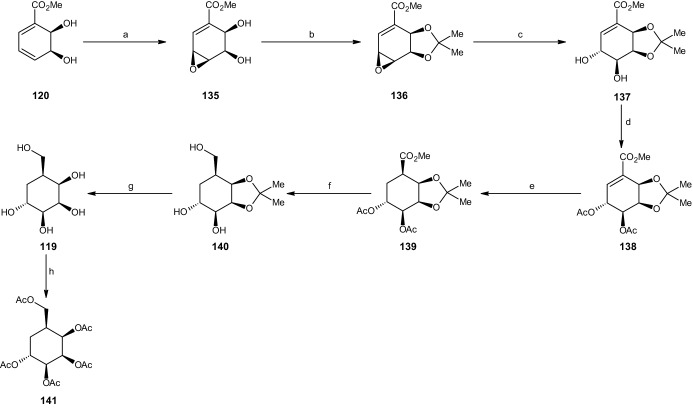
Synthesis of carba-α-l-talopyranose. Reagents and conditions: **a** MCPBA, CH_2_Cl_2_, 82% yield, **b** 2,2-DMP, *p*-TSA, 98% yield, **c** t-BuOH, H_2_O, pH 8 buffer, 68% yield, **d** Ac_2_O, C_5_H_5_N, 98% yield, **e** Rh/Al_2_O_3_, H_2_, EtOH, 83% yield, **f** LiAlH_4_, Et_2_O, 71% yield, **g** TFA, THF, H_2_O, 88% yield, **h** Ac_2_O, C_5_H_5_N, 82% yield

Finally, carba-*α*-l-talopyranose (**119**) was synthesized in seven steps (Scheme 27). The reaction started with the installation of an epoxide group at C4 and C5 of the cis-dihydrodiol derivative of methyl benzoate **120**. This was achieved via the addition of MCPBA in dichloromethane to give 82% cis-diol epoxide **135**. Compound **135** was protected by an acetonide (98% yield), followed by opening the epoxide ring using tert-butanol in water (pH 8 buffer) to produce 68% of cyclohexene trans-diol **137**. The two hydroxyl groups were then protected by acetylation, yielding 98% triester **138**. Through catalytic hydrogenation similar to previous reactions, compound **138** was transformed into the saturated ester **139** (83% yield). LAH was added to a Et_2_O solution to afford the trihydroxy acetonide-protected carba-*α*-l-talopyranose (**140**, 71% yield). The compound was deprotected using TFA and THF in water, yielding 88% of the desired carbasugar **119**. Furthermore, the compound was transformed into its penta-acetate derivative under the same conditions, with a yield of 82% (**141**) [51].

### Based on Benzoquinone

The final section covers the synthetic pathways for various unsaturated carbasugars, including streptol and gabosine, resulting from masked p-benzoquinone. Leermann et al. [16] explored the synthesis of several unsaturated carbasugars used in lectin-binding studies to determine the influence of sugar derivatives on enzyme inhibition and cancer therapy.

#### Synthesis of Dibromo Acetate Intermediates

**Scheme 28:**
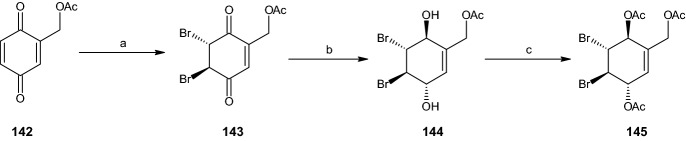
Synthesis of racemic intermediate **145**. Reagents and conditions: **a** Br_2_, CH_2_Cl_2_, 98% yield, **b** NaBH_4_, Et_2_O/H_2_O, 82% yield, **c** Ac_2_O, C_5_H_5_N, 51% yield

**Scheme 29:**
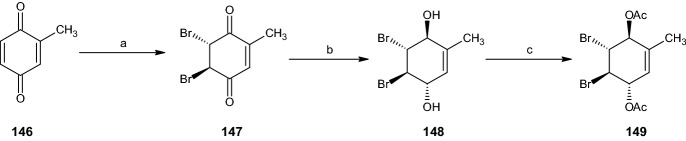
Synthesis of racemic intermediate **149**. Reagents and conditions: **a** Br_2_, CH_2_Cl_2_, 96% yield, **b** NaBH_4_, Et_2_O/H_2_O, 91% yield, **c** Ac_2_O, C_5_H_5_N, 60% yield

The starting materials for all pathways in this section were the two intermediates **145** and **149** formed from 2-acetoxymethyl benzoquinone (**142**) and 2-methyl benzoquinone (**146**), respectively (Schemes 28, 29). 2-Acetoxymethyl benzoquinone (**142**) was dibrominated regioselectively at the unsubstituted double bond, yielding 98% of dibromo acetate **143**. This compound was further reduced using sodium borohydride in diethyl ether and water (82% yield) and acetylated under the conditions described in previous sections to form the racemic dibromo acetoxymethyl diacetate intermediate **145** [16].

Under equal reaction conditions, 2-methyl benzoquinone (**146**) was transformed into the dibromo compound **147** (96% yield) and reduced to generate 91% of compound **148**. This compound was acetylated to form the dibromo methyl diacetate intermediate **149** with a 60% yield (Scheme 29) [16].

#### Synthesis of Unsaturated Penta- and Tetraols

**Scheme 30:**
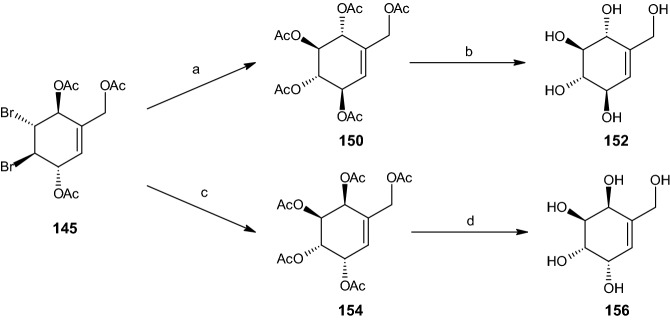
Synthesis of racemic diastereoisomers **152** and **156**. Reagents and conditions: **a** AgOAc, AcOH, Ac_2_O, 71% yield, **b** NaOMe, MeOH, 100% yield, **c** AgOAc, 90% AcOH, Ac_2_O, C_5_H_5_N, 35% yield, **d** NaOMe, MeOH, 82% yield

**Scheme 31:**
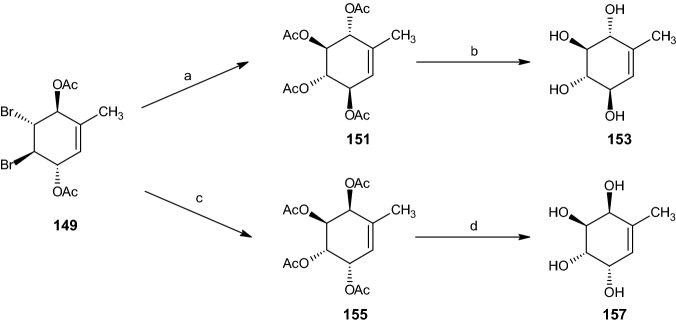
Synthesis of racemic diastereoisomers **153** and **157**. Reagents and conditions: **a** AgOAc, AcOH, Ac_2_O, 67% yield, **b** NaOMe, MeOH, 88% yield, **c** AgOAc, 90% AcOH, Ac_2_O, C_5_H_5_N, 32% yield, **d** NaOMe, MeOH, 88% yield

By utilizing Prévost conditions [52], silver acetate, acetic acid, and acetic anhydride, the dibromo acetoxymethyl diacetate intermediate **145** was transformed into penta-acetate **150** in 71% yield (Scheme 30). The entire yield of compound **150** was deacetylated via sodium methoxide in methanol, forming pentaol **152**. Similarly, the intermediate compound **149** was acetylated to give the tetra acetate **151** (67% yield) and deprotected to afford unsaturated rac-tetraol **153** (88% yield, Scheme 31) [16].

The penta-acetate **154** and tetra acetate **155** were obtained from intermediates **145** and **149**, respectively, through the use of Woodward conditions [50] (silver acetate in 90% aqueous acetic acid) alongside acetylation with acetic anhydride and pyridine, yielding 35% and 32% of compounds **154** and **155** (Schemes 30, 31), respectively. These two compounds were then converted into their corresponding alcohol compounds, rac-MK7607 (**156**, 82% yield) and **157** (88% yield) [16].

#### Synthesis of Streptol

**Scheme 32:**
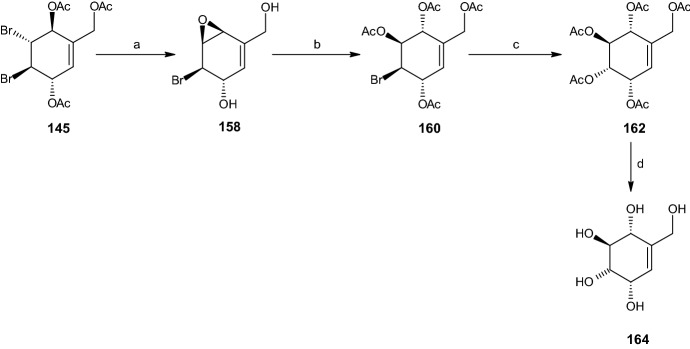
Synthesis of rac-streptol (**164**). Reagents and conditions: **a** LiOH, Et_2_O/MeOH, 83% yield, **b** *p*-TSA, H_2_O, Ac_2_O, C_5_H_5_N, 51% yield, **c** AgOAc, AcOH, Ac_2_O, C_5_H_5_N, 65% yield, **d** NaOMe, MeOH, 81% yield

Streptol (**164**) can be obtained from intermediate compound **145** in four steps, starting with the formation of an epoxide from the trans bromide and acetoxy group via addition of lithium hydroxide in Et_2_O and methanol, preserving relative configuration at C4 and C5 and yielding epoxide **158** (83% yield). Nucleophilic ring opening in water, followed by acetylation, yielded 51% of bromide diacetate **160**. Inversion and acetylation of the bromide group resulted in penta-acetate **162**. Deacetylation of **162** with methanolic sodium provided the desired rac-streptol (**164**) with an 81% yield (Scheme 32) [16].

#### Synthesis of Unsaturated Pentaol 165

**Scheme 33:**
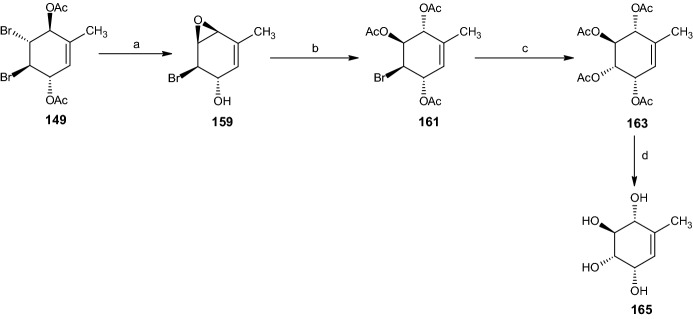
Synthesis of racemic tetraol **165**. Reagents and conditions: **a** LiOH, Et_2_O/MeOH, 72% yield, **b** CBr_4_, H_2_O, Ac_2_O, C_5_H_5_N, 37% yield, **c** AgOAc, AcOH, Ac_2_O, C_5_H_5_N, 38% yield, **d** NaOMe, MeOH, 88% yield

Similar to Scheme 32, the synthesis from compound **149** began with epoxidation to **159** (Scheme 33). Ring opening occurred through the addition of carbon tetrabromide in water, followed by acetylation, yielding 37% of the bromide triacetate compound **161**. Through bromide displacement by acetate, the authors obtained the methyl tetraol **165** (Scheme 33) with the same relative configuration as streptol (**164**) [16].

#### Synthesis of Unsaturated Pentaol 171

**Scheme 34:**
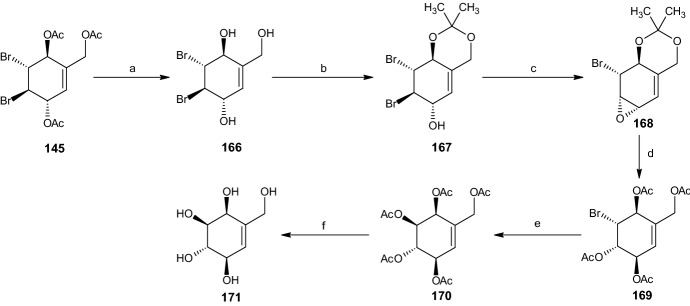
Synthesis of racemic pentaol **171**. Reagents and conditions: **a** K_2_CO_3_, MeOH, 100% yield, **b** 2,2-DMP, *p*-TSA, acetone, 100% yield, **c** NaOH, Et_2_O/H_2_O, 52% yield, **d** AcOH, H_2_O, Ac_2_O, C_5_H_5_N, 52% yield, **e** AgOAc, AcOH, Ac_2_O, C_5_H_5_N, 54% yield, **f** NaOMe, MeOH, 79% yield

The last pentaol compound was obtained in six steps (Scheme 34). Initially, intermediate **145** was deacetylated using potassium carbonate in methanol, resulting in dibromide diol **166**, whose vicinal hydroxyl groups were protected using 2,2-DMP and p-TSA in acetone, ultimately yielding 100% acetonide **167**. Epoxidation occurred via the addition of sodium hydroxide in Et2O and water with a 52% yield of epoxide **168**, followed by deprotection and acetylation comparable to those described in earlier pathways, yielding 52% of bromide tetra acetate **169**. The Woodward reaction conditions [47] ensured inversion and acetylation producing penta-acetate **170** in 54% yield. Finally, the deprotection of compound **170** yielded 79% of the desired unsaturated pentaol **171** [16].

## Summary

The present review highlights the success of carbasugar synthesis from non-sugar compounds. Due to their structural and configuration similarities with the desired products, the reactants used were able to deliver the products in few synthetic steps with comparatively high efficiency. These results suggest that future experiments may permit generation of the same products even faster and with higher yields. Due to the great diversity in the structures already known, the road is now paved for successful research in chemical and clinical medicine, including HIV and tumor treatment [14, 53, 54]. Due to the growing population and prevalence of common diseases, the importance of pseudo-sugar synthesis is greater than ever before. Ultimately, the synthetic routes described in this review offer information on already successful research and provide an impetus for the development of new discoveries in the field of carbasugars.
